# Evaluation of Antimicrobial Performance of Calcium Dihydroxide (Ca(OH)_2_) Coating on Ti for Potential Metallic Orthopedic Implant Applications

**DOI:** 10.3390/antibiotics14010091

**Published:** 2025-01-14

**Authors:** Harald Holeczek, Michael de Wild, Jasmine Ruegg, Philipp Gruner, Walter Moser, Olivier Braissant

**Affiliations:** 1 Medicoat AG, Almuesenacherstrasse 2, 5506 Mägenwil, Switzerland; h.holeczek@medicoat.ch (H.H.);; 2 Fraunhofer-Institut für Produktionstechnik und Automatisierung IPA, Nobelstr. 12, 70569 Stuttgart, Germany; 3 Fachhochschule Nordwestschweiz FHNW, Campus Muttenz, Hofackerstrasse 30, 4132 Muttenz, Switzerland; michael.dewild@fhnw.ch (M.d.W.);; 4 Atesos Medical AG, Schachenallee 29, 5000 Aarau, Switzerland; 5 Department of Biomedical Engineering, University of Basel, Hegenheimermattweg 167B/C, CH-4123 Allschwil, Switzerland

**Keywords:** calcium dihydroxide, Ca(OH)_2_, isothermal microcalorimetry, antimicrobial surfaces, orthopedic implants, orthopedic implant infections

## Abstract

**Background/Objectives:** Orthopedic implant infections are rare but represent a significant problem for patients, surgeons, and the healthcare systems. This is because these infections cause severe and persistent pain and, in some cases, may require revision of the implant, among other things. Thus, there is strong interest in the use of antimicrobial coatings on orthopedic implants. Here, we investigate electrochemically deposited Ca(OH)_2_ antimicrobial coating for its potential to be used on metallic orthopedic implants. **Methods:** A triphenyl tetrazolim chloride (TTC) assay and isothermal microcalorimetry (IMC) were used to determine the reduction in microbial activity on three sets of Ti parts (discs and screws): uncoated, coated with hydroxyapatite (HA), and coated with Ca(OH)_2_. **Results:** Using the TTC assay, a ~70% reduction in the growth of bacteria on Ca(OH)_2_-coated discs was found, and using IMC, bacterial growth on these discs showed a decreased rate and an increased lag phase up to 25 h. Each of these sets of results was statistically superior to the corresponding results obtained using the other sets of parts. **Conclusions:** The present results suggest that the Ca(OH)_2_ coating may have potential for use on metallic orthopedic implants.

## 1. Introduction

Calcium hydroxide (Ca(OH)_2_—commonly called “hydrated lime” or Portlandite) is known to be a potent antimicrobial agent [1]. It is a poorly soluble alkaline compound (K_sp_ = 5.02 × 10⁻^6^), with pH values > 12 in saturated solutions [2]. The high pH and the hydroxyl ions resulting from Ca(OH)_2_ dissociation are believed to lead to damage in microbial cytoplasmic membranes and DNA and contribute to protein denaturation [3]. Early in the 20th century, it was used to protect houses during an influenza pandemic [4,5] and was introduced in dentistry [6,7]. Nowadays, Ca(OH)_2_ is mostly used to prevent microbial outbreaks in animal houses, where it is used to protect walls against contamination or to disinfect manure and litter [8], as well as in new products such as antimicrobial paint [9]. In dentistry, many formulations relying on Ca(OH)_2_ are used during root canal treatment [10,11].

Despite the widespread use and acceptance of Ca(OH)_2_ as an antimicrobial agent, very few attempts have been made to use it as a coating on metallic implants. An electrochemical method of deposition produces a coating with thickness up to 20 µm [12]. Furthermore, the electrochemical deposition method also allows the addition of other ions in the coating solution used to deposit the Ca(OH)_2_ coating. Silver and copper may be added to the coating, leading to an additional antimicrobial effect of the coating [11]. Another method of coating deposition on Ti parts involves hydrothermal treatment of the part in a mixture of phytic acid and calcium hydroxide, which leads to the formation of thinner coatings (<1 um—[13]). Although this method leads to the deposition of calcium phosphate, traces of Ca(OH)_2_ and/or even more alkaline CaO might be present [14]. Thus, such coating cannot really be compared with Ca(OH)_2_ coating.

In addition to its antimicrobial properties, another potential benefit of Ca(OH)_2_ coating on an orthopedic implant is the prevention of aseptic loosening. Indeed, there is a growing body of evidence that questions the role that endotoxins play in aseptic loosening as well as the role of the undetected biofilms that produce them [15,16]. Many studies in this field have emphasized that Ca(OH)_2_ promotes alkaline hydrolysis of lipopolysaccharides (LPS) from Gram-negative bacteria and lipoteichoic acid (LTA) from their Gram-positive counterparts in vitro and in vivo [1,6,10,17,18,19,20]. For example, lipid A o-acyl hydrolysis under mild alkaline conditions is easily performed under laboratory conditions [21]. Considering the agreement on the reduction and hydrolysis of endotoxins by Ca(OH)_2_ [1,6,10,17], it is reasonable to assume that similar hydrolysis will take place in the presence of OH^−^ ions released by the Ca(OH)_2_ coating applied to an orthopedic implant. The possibility of hydrolysis of endotoxin at the surface of a Ca(OH)_2_ coating certainly deserves further studies in the context of orthopedic interventions.

In terms of safety and osseointegration of Ca(OH)_2_ coating on metallic orthopedic implants, electrochemically deposited Ca(OH)_2_ is considered safe, with animal studies showing no infection and good osseointegration [22]. In addition, in vitro tests with MG 63 cells and bacteria confirmed the antimicrobial activity of the coating. Ca(OH)_2_ coating deposited using electrochemical treatment was investigated only in vitro but showed promise in adhesion tests using human bone marrow mesenchymal stem cells and cell proliferation [12]. A Ca(OH)_2_ coating may also be deposited using an electrochemical method [23,24]. Such a coating consists mostly of Ca(OH)_2_ but also contains various amounts of hydroxyapatite (HA). The ratio of these two components may be modified by controlling the process parameters. The coating is more versatile than previously described coatings [25]. Little work has been reported on the antimicrobial performance of electrochemically deposited Ca(OH)_2_ coatings on metallic orthopedic implants. This is the subject of the present study, with the coatings being deposited on Ti6Al4V discs and screws with a pure Ti plasma-sprayed coating. The performance metrics were determined using conventional tri-phenyl tetrazolium chloride (TTC) assays and isothermal microcalorimetry (IMC). The latter method has proven to be a valuable tool in monitoring microbial activities at the surfaces of opaque and porous materials [26]. HA-coated materials commonly used in orthopedics were used as a comparison to assess the additional antimicrobial effect of Ca(OH)_2_-coated materials.

## 2. Results

### 2.1. Characteristics of Coating

**Figure 1:**
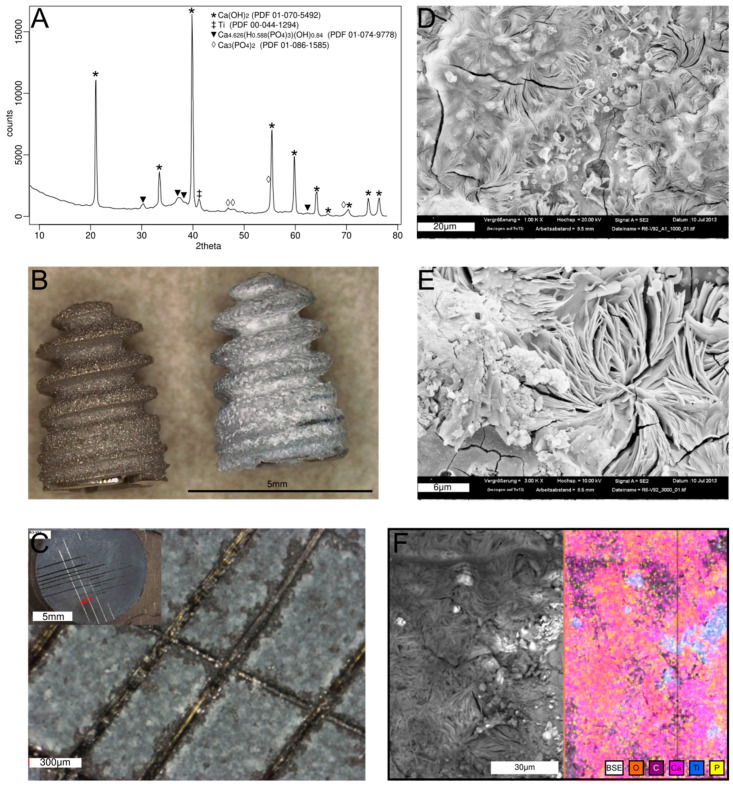
(**A**) X-ray diffractogram of the coating produced on a disc showing a strong crystalline Ca(OH)_2_ peak. (**B**) An uncoated titanium screw (left) next to a Ca(OH)_2_-coated screw (right). (**C**) Close-up view of a cross cut test performed on a coated disk (insert: full disc view). (**D**) Morphology of a the surface of a Ca(OH)_2_-coated disc. (**E**) High magnification view of the morphology of the surface of a Ca(OH)_2_-coated disc. (**F**) EDX mapping of a Ca(OH)_2_-coated showing predominantly Ca and O.

On both screws and discs, the coating appeared as a layer of white material that was composed mostly of Ca(OH)_2_ with detectable amounts of HA and traces of tri-calcium phosphates (Figure 1A,B) in X-ray diffractograms. The coating appeared reasonably adhesive to the surface as seen on a modified cross-cut test with ISO grading of 2 (less than 15% of the area affected by the cross cutting) (Figure 1C—[27]). Scanning electron microscopy revealed an open texture with cracks (Figure 1D,E), and energy-dispersive X-ray spectroscopy (EDX) analysis confirmed the composition of the coating comprising mostly Ca and O consistent with XRD data and expected composition of the coating (Figure 1F).

### 2.2. TTC Assay and Isothermal Microcalorimetry on Disk Samples

Both the TTC assay and the IMC results demonstrated that the Ca(OH)_2_ coating was antimicrobial and significantly decreased the growth of staphylococci. The TTC assay showed a 69% reduction in the microbial activity at the surface of Ca(OH)_2_-coated discs (Table 1) for both *S. epidermidis* (*p* < 0.05) and *S. aureus* (*p* < 0.05). No significant differences were observed between the uncoated and the HA-coated discs with respect to the growth of the two staphylococci tested on those surfaces (Table 1).

**Figure 2:**
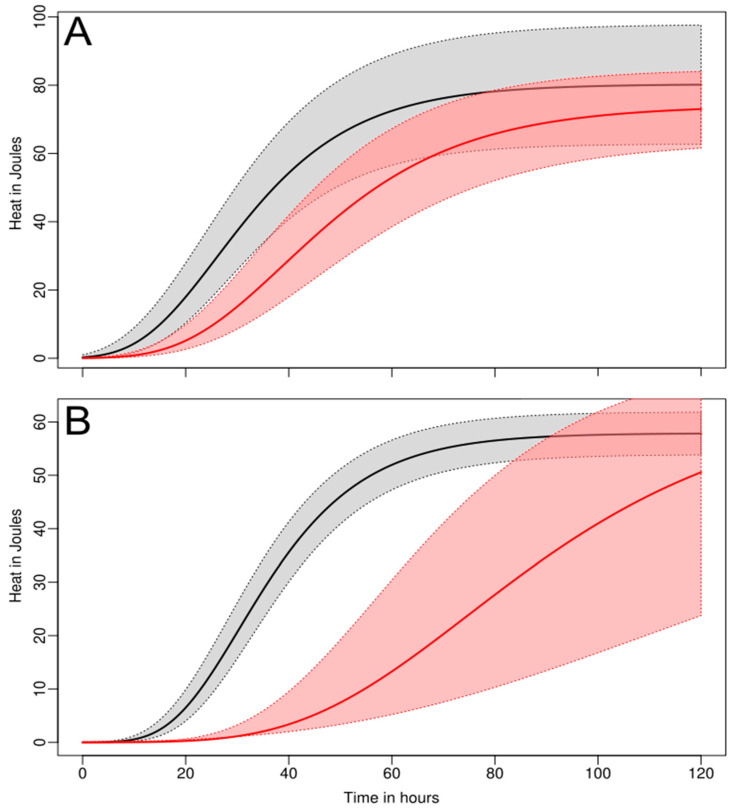
Growth curves of *S. epidermidis* (**A**) (10^5^ inoculum) and *S. aureus* (**B**) (10^3^ inoculum) at the surface of HA-coated (black) and Ca(OH)_2_-coated (red) discs. The growth curves were redrawn using the values from the growth parameters in Table 2. Plain curves indicate the average growth pattern and clear areas limited by dashed curves indicate the standard deviation.

Being a real-time method, IMC provided a more dynamic point of view. On discs incubated on a larger volume of agarized LB (10 mL), a clear difference could be seen between the two microbial strains tested. There was inhibition of the growth of *S. epidermidis* even at a larger inoculum (10^5^) with a decreased growth rate (μ) and an increased lag phase duration (λ) (Table 2 and Figure 2). On the contrary, at the same initial inoculum density, *S. aureus* did not show any significant growth inhibition (Table 2). However, at lower concentrations (10^3^), *S. aureus* was also inhibited, and its growth parameters also exhibited a lower growth rate (µ) and a longer lag phase (λ) (Table 2 and Figure 2). This emphasizes that the inoculation effect might be important for some microbial species and should be investigated in future studies.

### 2.3. Isothermal Microcalorimetry on Screws

**Figure 3:**
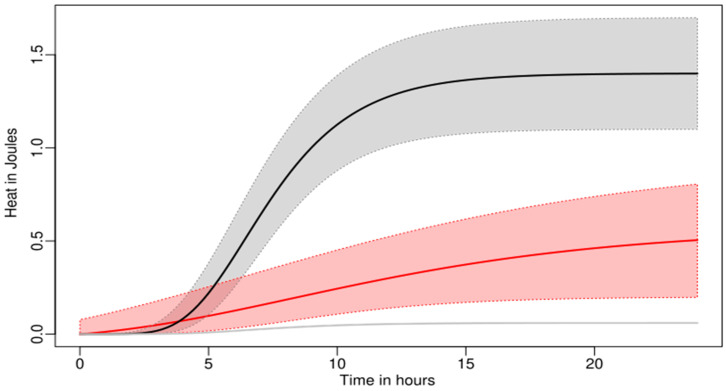
Growth curves of *S. epidermidis* (10^5^ inoculum) at the surface of HA-coated (black) and Ca(OH)_2_-coated (red) screws. The growth curves were redrawn using the values from the growth parameters in Table 3. Plain curves indicate the average growth pattern and clear areas limited by dashed curves indicate the standard deviation. (pure Ti data in Supplementary Figure S1).

There was a very strong inhibition of the growth of *S. epidermidis* on screws (Figure 3). This was expected from previous results, as the “dipping” inoculation method results in a small inoculate and because inoculum size effect was expected from the *S. aureus* data. In addition, the restricted volume (300 μL instead of 10 mL) likely emphasized the effect of OH^−^ ions released by the Ca(OH)_2_ coating. In this experiment, *S. epidermidis* growth was strongly inhibited on Ca(OH)_2_-coated screws compared to uncoated titanium discs (*p* < 0.05) and HA-coated discs (*p* < 0.05). The growth rate (µ) of *S. epidermidis* was strongly reduced (78% compared to HA and 59% compared to pure Ti coating). The lag phase (λ) was not extended and showed strong variability. However, with such a decrease in growth rate (µ), the Gompertz model used to evaluate the lag phase tended to compute shorter lag [19,20]. However, the difference in lag was not significant (Table 3 and Figure 3).

## 3. Discussion

**Figure 4:**
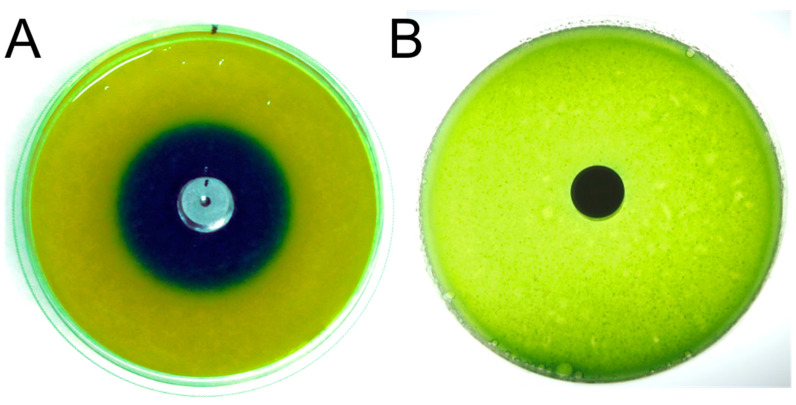
Abiotic experiment emphasizing the importance of buffer capacity on the pH rise induced by Ca(OH)_2_ coating. Effect of Ca(OH)_2_-coated ø15 mm discs in unbuffered agar 20 g/L (water agar) (**A**) and buffered agar media (Earle’s balanced salts added with bovine serum albumin 60 g/L to mimic serum protein levels—i.e., intended to have buffering capacity similar to that of blood or serum) (**B**). Both media contained the same pH indicator (Unisol 113—Macherey-Nagel—6% (*v*/*v*)).

The calcium hydroxide coating showed good capability to inhibit the growth of staphylococci, generally marked by a decreased growth rate (μ) and an increased lag phase duration (λ). The results presented here suggest that the sensitivity of the different strains to the OH^−^ ions released by the coating might differ markedly. In particular, our results with *S. aureus* at high (10^5^ cfu) and low (10^3^ cfu) inoculum densities suggest that there may be an inoculum effect. It must be noted that the shorter lag phase observed for Ca(OH)_2_-coated samples compared to control samples (i.e., HA) for *S. aureus* at higher density (10^5^ cfu) might be linked to the use of the Gompertz model potentially underestimating the lag at low growth rate (see details in [28,29,30]); however, the differences in lag are not statistically significant and should be interpreted with care. Still, the inoculates used here are very high compared to the amount of bacteria that might colonize an implant during surgery, as operating theaters/rooms are considered acceptable with an air quality of ca 90 CFU/dm^2^/hour [31]. Therefore, we expect that the coating will still provide acceptable protection against implant colonization and further infection. It is not unexpected to see such large variations in the effect of Ca(OH)_2_ inhibitory effect depending on the microorganisms but also on the assay used [32]. Also, depending on the method, the media, and the buffering capacity of the latter, the inhibition might appear very low (see below). As a consequence, the literature shows large discrepancies (and, sometimes, contradictions) with respect to the antimicrobial effect of Ca(OH)_2_ in vitro and the dynamic nature of such activity (i.e., time to microbial eradication) depending on the product, the vehicle, and many other parameters [33]. In our study, we used IMC to monitor the antimicrobial effect directly at the contact between the inoculum and the specimen, where the highest effect is expected. IMC provides the possibility to follow the growth or inhibition of the tested pathogen in real time on both hard, flat surfaces but also on non-flat surfaces (in our case, screws), making it an appealing method to test antimicrobial surfaces. In particular, in the case of Ca(OH)_2_ coating, we found that being able to monitor activity taking place between the surface of the coating and the media is crucial. Indeed, in preliminary abiotic experiments conducted without microbes, we found that in aqueous unbuffered or weakly buffered solutions (for example, water agar), Ca(OH)_2_ rapidly increases the pH of the surrounding medium or environments. However, in buffered solutions (for example, biological growth media and simulated biological fluids), this is not the case, and the pH changes as well as the antimicrobial effect are limited to the coated surface of the implant (Figure 4) or to zones very close to the coated surface. Thus, it is critical to choose methods able to assess antimicrobial effect at the surface of samples, especially when buffered microbiological media are used. In this context, using IMC to quantify the dynamics of bacterial growth and the antimicrobial effect at the very surface of the coated material is revealing [26].

Compared to end-point assays, such as the TTC assay used here, IMC offers the possibility of having time series without having to sacrifice and process a large number of specimens. Still, results of TTC staining of metabolically active bacteria and IMC show similar patterns, and both methods emphasize that a reduction in biofilm formation and activity at the surface of the Ca(OH)_2_ coating is clearly visible. Overall, the inhibitory effect at the surface of the Ca(OH)_2_ coating can be considered contact killing or contact inhibition, protecting the implant from large contamination for a certain duration. The timewise limited activity may also be beneficial for undisturbed long-term osseointegration of such surfaces. Whether the efficacy of contact killing in the highly buffered biological environment is of clinical relevance should be a subject for future preclinical studies.

Although the present study focused on a single set of coating conditions, with very small amounts of phosphate precipitated, the coating tested may be varied in terms of chemical composition, morphology, phase composition, and thickness by changing the concentration of citric acid during the coating process as well as the temperature, the electrical parameters, and treatment time. Indeed, the citric acid contained in the electrolyte formulation inhibits the formation of HA during the deposition process. The higher the citric acid concentration, the lower the amount of HA or other calcium phosphates found in the coating [34]. However, there is an important contrary effect. The deposition of the Ca(OH)_2_ out of the electrolyte at the cathode of the electrochemical cell is due to a rise in pH. The thicker the deposit gets, the smaller the current that can pass through the Ca(OH)_2_, as the latter acts as an insulator. At the same time, the citric acid in the electrolyte tends to dissolve the already deposited Ca(OH)_2_. Therefore, there is an antagonistic play between the pH-driven deposition and the chemically driven dissolution of the Ca(OH)_2_. This indicates that the coating can be modified by changing the electrolyte solution and potentially could also include other metals such as Ag or Cu (to improve antimicrobial properties—[11,26]) and/or Sr (to improve osteogenic properties—[35,36]). The performances of an implant coating should be seen within the framework of implant fabrication, where not only manufacturing cost but also the cost to the healthcare system are important factors. The coating method used in the present work, being driven by the electrochemically driven pH rise at the Ca(OH)_2_-coated surface, is a non-line-of-sight method, which allows coating of holes, pores, or undercuts of parts. This makes this method particularly suitable for coating rough Ti surfaces (pure Ti coating or Ti6Al4V) such as those found on many implants. The coating method combines ease, a high deposition speed (about 1 micrometer per minute), and a very high flexibility regarding the geometry of the parts that may be coated. The only requirements needed to coat a surface, even one that is geometrically complex, are a large enough electrolyte volume and a large electrode. Furthermore, it is also possible to apply the Ca(OH)_2_-coating to metallic Ti parts directly or on VPS-HA-coated ceramic surfaces. The equipment necessary for this process is simple and inexpensive. Additionally, the temperature needed is not too high, and the constituents of the electrolyte are very cheap chemicals. Therefore, this coating technology can be employed easily and be scaled according to meet the needs of size and number. Given these characteristics, the coating technology presented in this work may be promising for use in coating metallic implant surfaces.

The present study has some limitations. In particular, only *Staphylococcus* species were investigated. Other important pathogens, such as enterococci, streptococci, and pseudomonads, should also be investigated to provide a complete image of the efficacy of such coating. Still, as staphylococci represent the vast majority (up to 70%) of orthopedic device-related infections [37,38,39], we believe that the results presented here provide meaningful evidence of the usefulness of such coating. Some key properties have not been investigated yet. Among those, the small size of the parts produced in this study only allowed for a modified scratch test and a limited assessment of the adhesion of the coating to the metallic part. Therefore, adhesion should be investigated in more detail; however, the preliminary data presented here suggest good adhesion, especially considering that the coating is expected to dissolve/resorb in the patient with time. Indeed, the dissolution kinetics should be investigated as well to estimate the duration of the protection of the patient against infections. In addition, considering so-called aseptic loosening, the hydrolysis of LPS and LTA at the implant surface should be investigated, as this feature would provide a strong benefit to such coating. Finally, new technologies to deliver antimicrobials directly at infection sites are investigated (see examples in references [40,41]) and should be compared with the present coating. However, the blood–bone barrier remains difficult to penetrate for antimicrobials, making the application of coating as preventive measures quite advantageous, especially considering that the price of such coating is expected to be low compared to other technologies (see above). Also, some of these technologies, such as applying nanostructure to the coating, may not be compatible with the coating process and, thus, should be investigated as well.

## 4. Materials and Methods

### 4.1. Coating Procedures for Disk and Screws Used

Sand-blasted parts (ø10 mm Ti6Al4V discs and ø3.5 mm screws) were vacuum plasma spray coated with pure titanium. After this initial coating, they were thoroughly degreased in an alkaline solution and rinsed with de-ionized water. Furthermore, they were placed for 3 min in acetone in an ultrasound bath and rinsed again with deionized water. Following this step, they were conditioned in citric acid for 30 s and placed in an electrolyte that consisted of 84 mM calcium nitrate, 25 mM ammonia hypophosphate, and 50 mM citric acid. During this step, discs were maintained in a PVC holder that allowed the electrolyte to be in contact with only one face of a disc. The holder was placed in the center of a cylindrical anode made of Pt-coated Ti. The electrolyte was held at a temperature of 50 °C and thoroughly stirred to support the detachment of any gas bubbles from the active surfaces during the coating process. A current density of 240 mA/cm^2^ was maintained throughout the deposition process of 20 min during which the potential was held at 100–120 V, depending on the part being coated. After the end of the deposition time, the part was removed from the electrolyte still under the electric potential to prevent any dissolution of the coating. Discs were briefly rinsed with de-ionized water and then dried in hot air before dismounting from the holder. Coated parts were subjected to X-ray diffraction (XRD) analysis (Philips PW3710 powder diffractometer, Philips Analytical, Almelo, The Netherlands) and scanning electron microscopy (SEM, Gemini 300 series, Zeiss and Hitachi TM-1000 equipped with a Quantax 70 (Bruker, Billerica, MA, USA) EDX probe) to determine the composition, morphology, and thickness of the deposited coating. In addition, screws were imaged with a Leica DMV6 microscope (Leica Microsystems, Heerbrugg, Switzerland). The coating mechanical stability was assessed on coated disks using a modified cross-cut test. Briefly, due to the small size of the coated discs, a fresh cutter blade was used to make a cross-cut on a surface. Adhesive tape was then applied onto the marked grid and removed with an even peeling movement. The adhesion was quantified according to the ISO 2409:2013 scale [27]. Uncoated (i.e., only pure Ti coating) and vacuum plasma spray HA-coated samples were used as comparisons.

### 4.2. Microorganisms and Growth Conditions

We used *Staphylococcus epidermidis* (ATCC 49461) and *Staphylococcus aureus* (ATCC 25923) because these two microorganisms are frequently isolated in clinical investigations of infections of metallic orthopedic implants [37,39]. Stocks of the bacteria in Luria broth (LB) medium added with 20% (*v*/*v*) glycerol were stored at −80 °C. Prior to each test, an aliquot was unfrozen and used as inoculum for an overnight culture performed in LB medium at 37 °C. The optical density (OD_600_) of the medium was measured, and appropriate dilutions were made to reach the target concentration for further tests (see below).

### 4.3. Triphenyl Tetrazolim Chloride (TTC) Assay on Discs

Coated and uncoated discs were placed in a 24-well plate, and 2.5 mL of LB medium (LB—tryptone (pancreatic digest of casein) 10 g/L, yeast extract 5 g/L, NaCl 10 g/L: Sigma Aldrich, St. Louis, MO, USA) inoculated at a target concentration of 10^5^ CFU·mL^−1^ were added to each well. The plates were then incubated at 37 °C for 24 h. After incubation, the medium was removed and the discs were washed 3 times in PBS to remove non-adherent bacteria. Then, 2.5 mL of LB medium containing 0.4% (*w*/*v*) 2,3,5-triphenyl-tetrazolium chloride were added to each well. After 2 h of incubation and the development of a strong reddish color typical of formazan, the discs were washed 3 times in PBS, and the formazan formed on the disc was extracted in 100% ethanol. Finally, ethanol extract was centrifuged to remove cell debris, and the absorbance of the supernatant was obtained at 490 nm. The measurements were performed using at least 4 replicates. Blanks were made using sterile uninoculated discs with only pure Ti coating.

### 4.4. Measurement of the Metabolic Activity Close to the Ca(OH)_2_ Layer by Isothermal Microcalorimetry

#### 4.4.1. Isothermal Microcalorimetry of Discs

The measurement of the metabolism of bacteria growing close to a disk was performed as described previously [35,36]. After sterilizing the discs by exposure to UV light for 30 min with intermittent rotations, 10 µL of a diluted overnight culture was used to inoculate the Ca(OH)_2_ surface of the disc with of 10^5^ bacterial cells (alternatively, when little effect was observed, the experiment was repeated with an inoculum of 10^3^ bacteria as cell aggregates and clumps forming at higher concentrations were hypothesized to protect the cells). Then, the disc was placed in a microcalorimetric vial containing 10 mL of solid Luria agar (tryptone (pancreatic digest of casein) 10 g/L, yeast extract 5 g/L, NaCl 10 g/L, agar 15 g/L) with the inoculated Ca(OH)_2_ side facing the medium. Slight pressure was applied on the disc for 10 s to ensure close contact between the coating and the medium. Finally, the microcalorimetric ampoule was closed and placed in the calorimeter (TAM air calorimeter; Waters/TA, New Castle, DE, USA), with an appropriate reference ampoule containing the same volume of uninoculated Luria agar. The metabolic heat produced was recorded from the beginning of the test until it returned to baseline level. Blanks were performed using sterile, uninoculated, and uncoated discs.

#### 4.4.2. Isothermal Microcalorimetry of Screws

After sterilizing the screws by exposure to UV light for 30 min with intermittent rotation, they were placed in a recipient containing 200 µL of overnight culture for 1 min. The “dipping” inoculation method was chosen because of the difficulties in spreading the inoculum homogeneously at the surface of a screw (compared to a disc). The screws were then briefly rinsed in PBS, and the excess liquid was removed by placing the screw on a sterile tissue. The screws were placed in a calorimeter insert filled with 300 μL of solid Luria agar (2% agar) using an appropriate screwing tool. Blanks were performed using uninoculated and uncoated screws. Additional blanks were performed using Ca(OH)_2_-coated screws as the pH rise in the small volume resulted in CO_2_ binding and heat production. This series of blanks was used to subtract the heat of the CO_2_ reaction with OH^−^ ions from that released from inoculated specimens.

### 4.5. Statistical Analysis

Calorimetric data were integrated and fitted with the Gompertz growth model, as described previously [28,29,30,42,43]. The growth rate (μ), lag phase (λ), and total heat produced (Q) were extracted. The TTC assay data were expressed as percentage of control (PS). After normality of datasets was tested using the Shapiro–Wilk test, normally distributed datasets were compared using *t*-test or ANOVA. Non-parametric tests (Kruskal–Wallis and Mann–Whitney U-test) were used in cases where the datasets being compared were not normally distributed. All calculations were performed with R (3.6.3) and the Grofit package [43,44].

## 5. Conclusions

Using isothermal microcalorimetry and tetrazolium salts reduction assays, we found that the Ca(OH)_2_ coating on pure Ti-coated parts (discs and screws) demonstrated a contact-killing/inhibiting effect on *S. aureus* and *S. epidermidis*. The antimicrobial effect was clearly visible when comparing the Ca(OH)_2_-coated samples with HA-coated reference material. The coating method is simple and inexpensive. Thus, this coating may have potential to be used on metallic orthopedic implants. The coating is simple to prepare and fits a wide variety of metallic implant shapes. The cost of production for such coating is expected to be low, thus putting minimal financial pressure on patients, practitioners, or healthcare systems. However, the clinical relevance of the contact killing effect of the Ca(OH)_2_ coating with its endotoxin-hydrolyzing properties needs to be investigated.

## Figures and Tables

**Table 1:** Measure of growth of metabolically active biofilm on discs determined using the TTC assay. The activity of each sample was normalized to the growth on an uncoated disc (i.e., pure Ti coating) (100%).

	Coating	% Growth	*p*-Value ^1^	n ^2^
***S. aureus***	Pure Ti coating	100.0 ± 32.5%		12
	HA	91.0 ± 16.5%	0.50	4
	Ca(OH)_2_	30.2 ± 8.0%	<0.05	12
***S. epidermidis***	Pure Ti coating	100.0 ± 41.2%		12
	HA	81.9 ± 6.7%	0.38	4
	Ca(OH)_2_	30.4 ± 0.9%	<0.05	4
**Blanks**	Pure Ti coating	0.0 ± 0.0%	<0.05	12

^1^ Student *t*-test. ^2^ number of replicates.

**Table 2:** Growth parameters (growth rate (μ) and lag phase duration (λ)) extracted from isothermal calorimetry measurements for *S. epidermidis* (10^5^ cfu) and *S. aureus* (at high 10^5^ cfu and low 10^3^ cfu inoculum) on the surface of discs placed at the surface of 10 mL of agar medium.

	Coating	μ [J·h^−1^]	*p*-Value	λ [h]	*p*-Value	n ^2^
***S. epidermidis***	HA	1.98 ± 0.34		11.1 ± 3.1		3
(10^5^ CFU)	Ca(OH)_2_	1.41 ± 0.33	<0.05	19.6 ± 3.8	<0.05	4
	Blanks ^1^	0.00 ± 0.00		NA		3
***S. aureus***	HA	2.96 ± 0.89		18.1 ± 8.8		3
(10^5^ CFU)	Ca(OH)_2_	2.67 ± 0.34	0.64	11.0 ± 5.7	0.30	4
	Blanks ^1^	0.00 ± 0.00		NA		3
***S. aureus***	HA	1.60 ± 0.12		17.1 ± 2.3		3
(10^3^ CFU)	Ca(OH)_2_	0.74 ± 0.39	<0.05	42.6 ± 9.5	<0.05	4
	Blanks ^1^	0.00 ± 0.00		NA		3

^1^ Uninoculated disks. ^2^ Number of replicates.

**Table 3:** Growth parameters (growth rate (μ) and lag phase duration (λ)) extracted from isothermal calorimetry measurements for *S. epidermidis* (10^5^ cfu) at the surface of screws inserted in 300 µL agar medium. Measurements took place in a Symcel Calscreener (see Section 4 for details).

Material	μ [J·h^−1^]	*p*-Value ^1^	λ [h]	*p*-Value ^1^	n
Pure Ti coating	0.12 ± 0.02	<0.05 ^2^	4.3 ± 0.3		5
HA coating	0.22 ± 0.03	<0.05 ^2^	4.1 ± 0.6	0.68	5
Ca(OH)_2_ coating	0.05 ± 0.02	<0.05 ^2^	0.9 ± 3.7	0.06	5
Blanks ^3^	0.01 ± 0.00	<0.05 ^2^	NA	NA	3

^1^ Student *t*-test. ^2^ Also significantly different from each other. ^3^ Uninoculated disks.

## Data Availability

The raw data supporting the conclusions of this article are available from the authors on request.

## Supplementary Materials

The following supporting information can be downloaded at: https://www.mdpi.com/article/10.3390/antibiotics14010091/s1, Figure S1: Growth of *S. epidermidis* (A) (10⁵ inoculum) at the surface of uncoated (green), HA coated (black) and Ca(OH)_2_ coated (red) screws. The growth curves were redrawn using the values from the growth parameters in Table 3. Plain lines indicate the average growth pattern, clear areas limited by dashed lines indicate the standard deviation.
