# Supplementary material for: Evaluation of Antimicrobial Performance of Calcium Dihydroxide (Ca(OH)2) Coating on Ti for Potential Metallic Orthopedic Implant Applications
Source: Antibiotics (Basel). 2025 Jan 14;14(1):91. doi: 10.3390/antibiotics14010091 (PMC11761600; doi:10.3390/antibiotics14010091)
Supplement: Supplementary file 1 [file antibiotics-14-00091-s001.zip › antibiotics-3240631-supplementary.pdf]

antibiotics

Article

Evaluation of Antimicrobial Performance of Calcium Dihydroxide ($Ca(OH)_2$) Coating on Ti for Potential Metallic Orthopedic Implant Applications

Harald Holeczek [1,2], Michael de Wild [3], Jasmine Ruegg [3], Philipp Gruner [1], Walter Moser [4] and Olivier Braissant [5,*]

1 Medicoat AG, Almuesenacherstrasse 2, 5506 Mägenwil, Switzerland; h.holeczek@medicoat.ch (H.H.);
2 Fraunhofer-Institut für Produktionstechnik und Automatisierung IPA, Nobelstr. 12, 70569 Stuttgart, Germany
3 Fachhochschule Nordwestschweiz FHNW, Campus Muttenz, Hofackerstrasse 30, 4132 Muttenz, Switzerland; michael.dewild@fhnw.ch (M.d.W.)
4 Atesos Medical AG, Schachenallee 29, 5000 Aarau, Switzerland
5 Department. of Biomedical Engineering, University of Basel, Hegenheimermattweg 167B/C, CH-4123 Allschwil, Switzerland
* Correspondence: olivier.braissant@unibas.ch (O.B)

Academic Editor: Marc Maresca

Received: 18 September 2024
Revised: 7 January 2025
Accepted: 10 January 2025
Published: 14 January 2025

Citation: Holeczek, H.; de Wild, M.; Ruegg, J.; Gruner, P.; Moser, W.; Braissant, O. Evaluation of Antimicrobial Performance of Calcium Dihydroxide ($Ca(OH)_2$) Coating on Ti for Potential Metallic Orthopedic Implant Applications. *Antibiotics* **2025**, *14*, x. https://doi.org/10.3390/xxxxx

Copyright: © 2025 by the authors. Submitted for possible open access publication under the terms and conditions of the Creative Commons Attribution (CC BY) license (https://creativecommons.org/licenses/by/4.0/).

antibiotics

MDPI

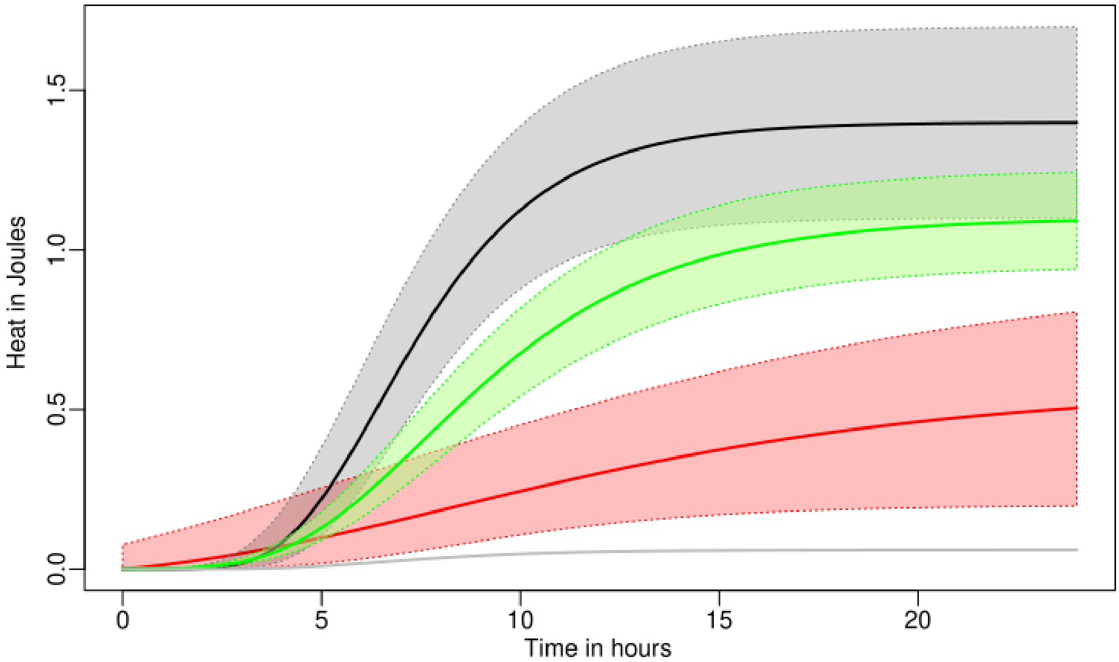

Supplementary Figure S1. Growth of *S. epidermidis* (A) (10^5 inoculum) at the surface of uncoated (green), HA coated (black) and $Ca(OH)_2$ coated (red) screws. The growth curves were redrawn using the values from the growth parameters in table 3. Plain lines indicate the average growth pattern, clear areas limited by dashed lines indicate the standard deviation.
